# The evolution of cooperation in signed networks under the impact of structural balance

**DOI:** 10.1371/journal.pone.0205084

**Published:** 2018-10-08

**Authors:** Xiaochen He, Haifeng Du, Meng Cai, Marcus W. Feldman

**Affiliations:** 1 Center for Administration and Complexity Science of Xi’an Jiaotong University, Xi’an, Shanxi Province; 2 School of Economics and Management, Xidian University, Xi’an, Shanxi Province; 3 Department of Physics, Boston University, Silber Way, Boston, United States of America; 4 Morrison Institute for Population and Resource Studies, Stanford University, Stanford, United States of America; Southwest University, CHINA

## Abstract

Structural balance plays an important role in the dynamics of signed networks. Based on structural balance, we generalize the evolution of cooperation in signed networks. Here we develop a new simulation model to study the impact of structural balance on the evolution of cooperation in signed networks. The simulation shows that cooperation prevails when an individual has a higher probability of adjusting the signs of its relations. We also find that structural balance forces the coexistence of cooperators and defectors, while the initial attributes of networks have little impact on the evolution of cooperation in the presence of structural balance, although they have a strong effect on the evolution of structural balance.

## Introduction

Cooperation plays an important role in human societies, but its evolution in a population consisting mainly of selfish individuals is difficult to explain [1]. Game theory has laid a foundation on which to study this puzzle. In conventional evolutionary game theory, cooperators will be wiped out by defectors in the well-mixed prisoner’s dilemma (PD) game [2–5]. The inevitability of defection is relaxed in the snowdrift and stag-hunt games, which offer more support for cooperative individuals [6–9]. Moreover, structured populations can give different results from the well-mixed case, and when the game is played in sparse networks, cooperation may spread [10–14]. Remarkably, complex networks, such as scale-free networks, can promote cooperation, owing predominantly to their inherent heterogeneity [15–21]. The impact of network structure has also been studied for other evolutionary games such as the rock-paper-scissors game [21, 22] or the public goods game [23–25]. Natural additional model features including reward and costly punishment [25, 26], variation in strategy transfer capability [27], coevolution of teaching activity [28], noise [29–31], memory effects [32], different populations with neutral payoff [33] and a preferential selection mechanism [34] have been employed in these studies. In Y. Li et al. [35], a player who has larger payoff shares some his payoff with his comparatively poorer neighbors, which also can promote the evolution of cooperation.

Several studies have focused on the evolution of cooperation in adaptive networks in which the social ties can be adjusted. These models give similar results: cooperation prevails when individuals have a higher probability of adjusting their social ties [36–42]. Q. Li et al. studied the coevolution of quantum on weighted random networks, in which individuals can rewire their links in order to improve their payoffs [39]. Szolnoki et al. introduced a new evolutionary model in which existing links are deleted whenever an individual shifts its strategy or its degree exceeds a threshold value, and new links are randomly added after several iterations [40, 42]. Pinheiro et al. draw a link between the individual interactions and population-wide dynamics in the process of evolution [38]. However, these models are constructed on 0–1 networks, where the effect of edges’ existence is stressed, while the impact of different attributes of edges, such as their positive and negative signs, has been ignored [43]. In the real world, interactions between individuals are not only accompanied by affiliation, trust or cooperation, but are regularly subject to disagreement, controversy or sometimes outright conflict [44]. Networks that have both positive and negative relations are called signed networks, where the sign “+” represents a positive relation, while “–” represents a negative relation [45]. These signed relations can simply signal cooperative or non-cooperative intentions, and thus provide information that individuals may use to adjust their strategies or their social ties in the repeated game [43]. Righi and Takacs [1, 46] and Simone and Karoly [43] generalized the PD model in signed networks, and first introduced relational signs to summarize affect and emotions between interacting partners. In their research, the individual strategies include not only cooperation or defection, but also an emotional conditional strategy; that is, cooperation with individuals who are linked with positive relations but defection with those who are linked with negative relations. Although they considered the impact of different signs of relations on the emotion of individual strategies, they ignored the impact of different signs among relations on the payoff from the game. Actually, the payoff can be quite different the presence of positive and negative relations [47], e.g., the PD payoff matrix could be different with different relation signs. Moreover, some important properties of signed networks, such as structural balance, were not considered, and these may strongly affect the PD model.

The theory of structural balance plays an important role in the dynamic evolution of signed networks [48]. The PD model considers rational choice by individuals when they interact with others, while structural balance focuses on emotional choice. Therefore, combining structural balance with the PD model may produce a more natural set of choices. Heider first proposed the definition of structural balance to detect the origin of conflicts in networks [49], and this definition was generalized later by Cartwright and Harary based on graph theory [50]. Similarly to the evolution of cooperation based on the PD model, a series of studies has focused on the dynamic evolution of structural balance [51–57]. Du et al. [57] analyzed structural balance in fully signed networks, where the node attribute could also be represented by signs “+” or “–”, and the network evolution was based on rules for simultaneously adjusting signs of relations and nodes. Some rules in this model can be also applied to the dynamic evolution of networks based on PD, because both models focus on the adjustment of nodes and relations.

The new combined model may be more realistic, because the social network’s relations are not homogeneous, and a group of people with different relation attributes playing the PD game can represent a wider range of real world situations. One of the applications is how the government and civilians reach a consensus concerning a big program, where different ideas and different kinds of relations exist. In this paper, we generalize the PD model proposed by Santos et al. [36] and Pinheiro et al. [38] in signed networks, based on the theory of structural balance. We aim to find how cooperation evolves in a signed network, and how the edges with negative relations affect the evolution. In the following, we analyze the impact of structural balance on the PD model, and propose a new evolutionary model for playing the game in signed networks. Then we discuss our experimental results.

### Structural balance and the PD model

**Fig 1:**
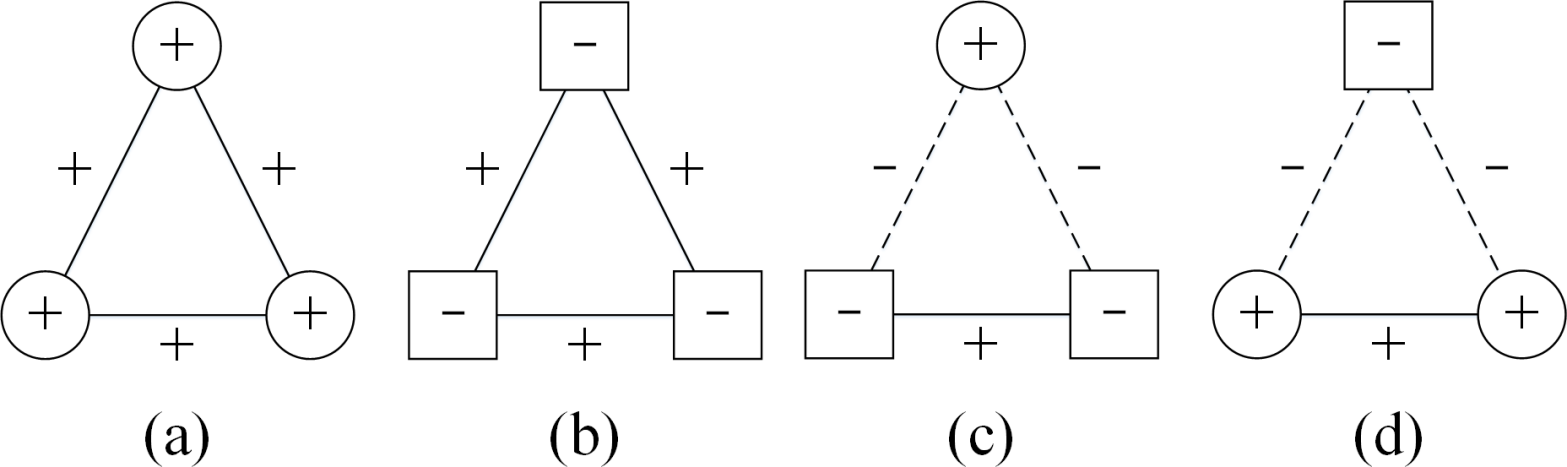
All balanced triangles in fully signed networks. The solid lines with ‘+’ denote positive relations, the dashed lines with ‘–’ denote negative relations, the circle nodes with ‘+’ denote the +1 nodes that represent one point of view, while the square nodes with ‘–’ denote –1 nodes that represent the opposite point of view. According to Heider’s definition of structural balance [49], the balanced triangles consist of zero or two negative edges, which complies with the logic “my friend’s friend is my friend” and “my friend’s enemy is my enemy”. According to the structural balance in fully signed networks [57], nodes with same attributes should be connected by a positive edge, while pairs of nodes with opposite signs should be connected by a negative edge. Thus there are only four kinds of balanced triangles.

**Fig 2:**
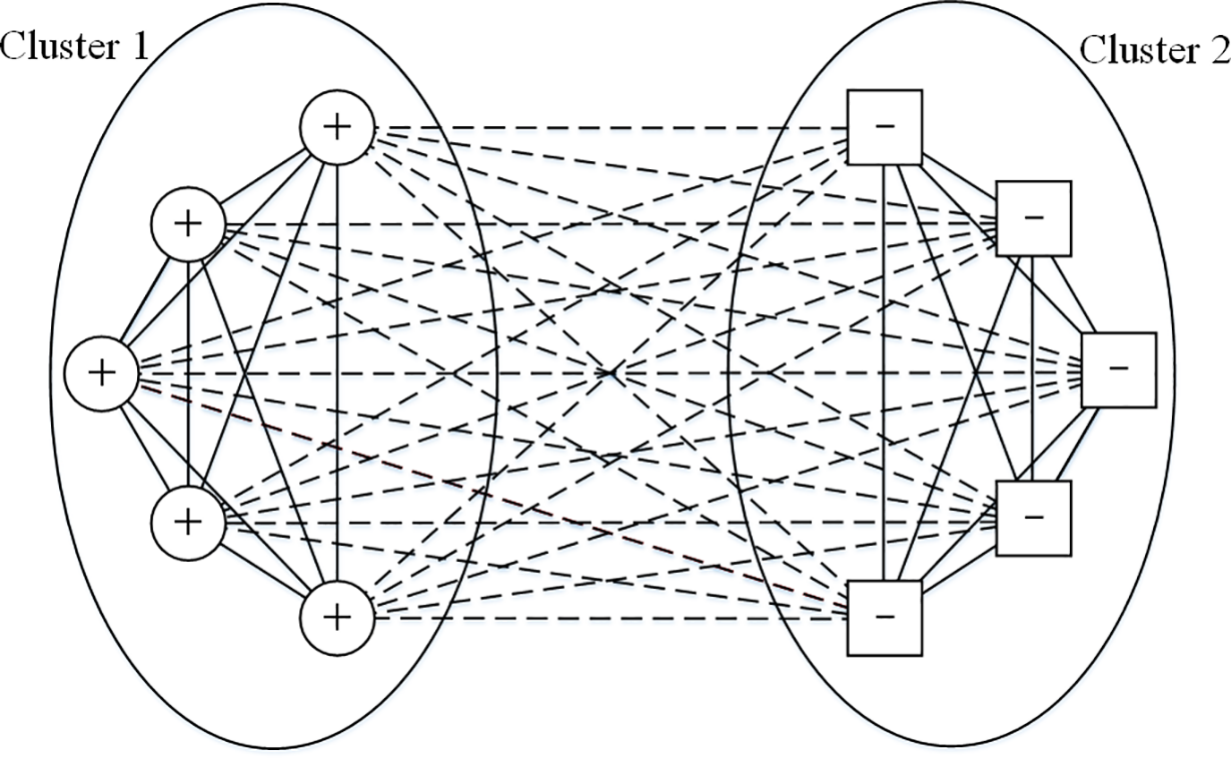
A fully balanced network from global viewpoint. The solid lines denote positive relations; the dashed lines denote negative relations; the circle nodes with ‘+’ denote the +1 nodes, while the square nodes with ‘–’ denote –1 nodes. From a global view, a balanced network can be divided into two groups, within which nodes have the same attribute and are connected by positive edges, and between which nodes have different attributes and are connected by negative edges.

According to the definition of structural balance in fully signed networks, there are only four kinds of balanced triangles, as shown in Fig 1 [57]. From a whole-network view, a balanced structure can be formed as shown in Fig 2, which assumes the principle of homophily, namely, individuals tend to imitate their friends’ attributes, while having different attributes from those of their enemies [58, 59]. However, this assumption is not entirely appropriate for the PD model, because the node strategy (cooperation or defection) in the PD model has its distinct payoff, while the structural balance model assumes equality of different node attributes. For example, structural balance assumes the effect of three “+” nodes connecting positive links with each other equals that of three “–” nodes connecting positive links with each other, and the three nodes no matter with the attribute “+” or “–” will be balanced if they connect positive links with others who have the same attribute, and they will not have any payoff. However, the effect of three cooperators connecting positive links with each other is quite different from that of three defectors connecting positive links with each other. Since players in the PD model have their own payoffs, all three players will receive rewards if they are cooperators, and they will be punished if they defect. The motivation of changing node strategies or changing relations can be quite different between these two situations. Thus, the form of these triangles should be altered when we study the PD model in signed networks.

**Fig 3:**
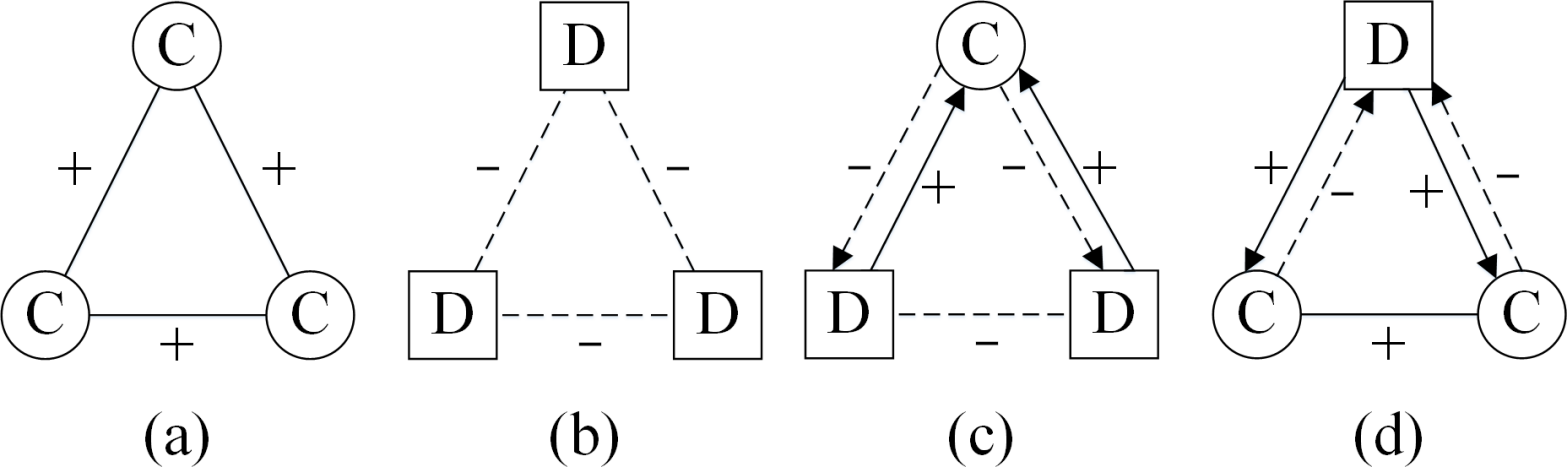
All ideal triangles for PD model in signed networks when not considering of structural balance. The solid lines with ‘+’ denote positive relations; the dashed lines with ‘–’ denote negative relations; the circle nodes with ‘C’ denote the cooperators; the square nodes with ‘D’ denote defectors. In these triangles, everyone tends to connect positive edges with cooperators, while they connect negative edges with defectors.

In Refs. [36, 38], everyone wants to connect with cooperators, while keeping a distance from defectors. Based on this rule, the form of ideal triangles for the PD model in signed networks can be displayed as in Fig 3. For a triangle consisting of three cooperators as shown in Fig 3(A), the nodes want to make friends with each other, because they can benefit from each other; In Fig 3(B), all game players are defectors, in which case they will not believe each other, and have negative relations with each other; for a triangle consisting of one cooperator and two defectors, as shown in Fig 3(C), the two defectors connect negatively with each other, but want to make friends with the cooperator. However, afraid of being cheated, the cooperator tends to have negative relations with the two defectors; In Fig 3(D), the two cooperators connect positively with each other because they can benefit from each other, and do not make friends with the defector, although the defector is willing to make friends with them.

**Fig 4:**
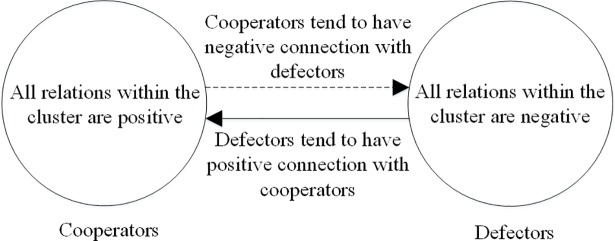
A global view of the ideal network structure for PD model in signed networks when structural balance is ignored. In this structure, cooperators that are positively connected with each other tend to have negative connections with defectors, while defectors that are negatively connected with each other tend to have positive connections with cooperators. This structure includes all the kinds of triangles shown in Fig 3.

From a global view, when all triangles are the ideal ones shown in Fig 3, the whole network structure can be divided into two clusters: the clusters of cooperators and defectors as shown in Fig 4. Within the cluster of cooperators, all relations are positive, while within the cluster of defectors, all relations are negative. Between these two clusters, cooperators tend not to want connections with defectors. However, the triangles in Fig 3(C) and Fig 3(D) are not stable because cooperators and defectors have opposite opinions about their relation signs. So when the whole network structure becomes stable, all triangles will evolve to take the form in Fig 3(A), where all players become cooperators and all relations become positive; or all triangles will evolve to take the form of Fig 3(B), where all players become defectors and all relations become negative.

**Fig 5:**
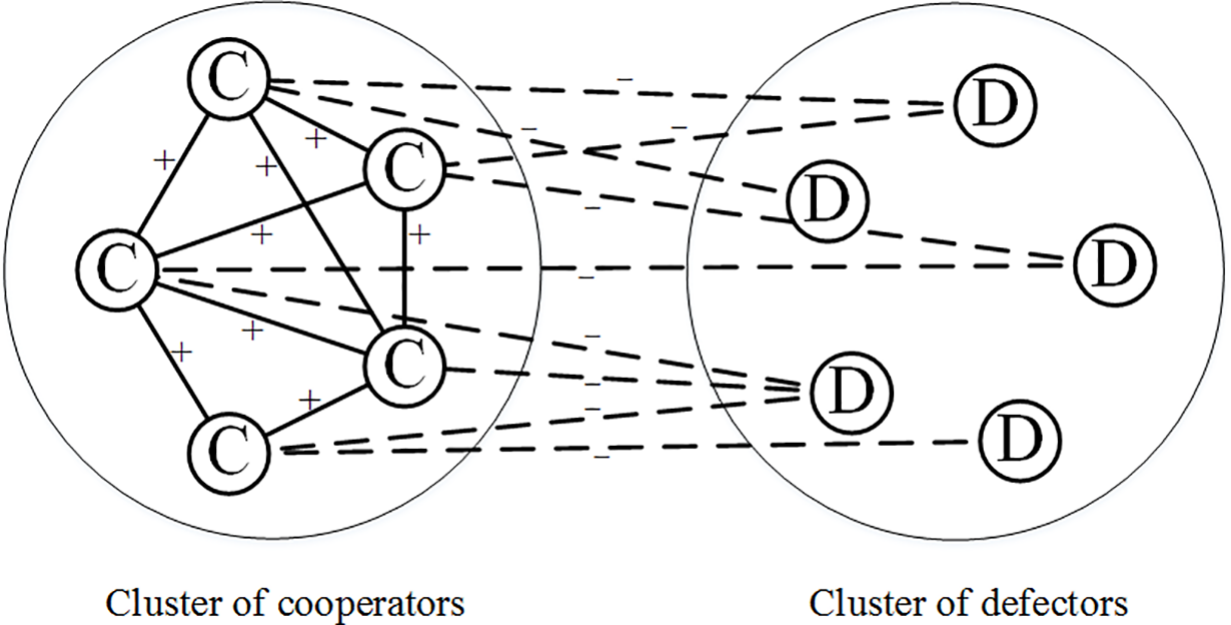
A global view of the ideal network structure for PD model in signed networks when considering structural balance. The solid lines with ‘+’ denote positive relations; the dashed lines with ‘–’ denote negative relations; circle nodes with ‘C’ denote the cooperators; square nodes with ‘D’ denote defectors. In this structure, cooperators with positive connections connect negative edges with those defectors with no edges connected. This is a balanced structure, and if we regard ‘C’ as ‘+’ and ‘D’ as ‘–’, then all the triangles in this structure will be of the form in Fig 1(A) and Fig 1(D).

However, the triangles in Fig 3(B) and Fig 3(C) are not balanced according to the definition of structural balance in fully signed networks, because the negative relation connecting two defectors contributes to the global imbalance [57]. Moreover, under the impact of structural balance, the triangle in Fig 3(D) should be transformed to the form in Fig 1(D), when the network becomes stable. As a result, when we consider the impact of structural balance, there should be only two kinds of balanced triangles, namely three cooperators having positive relations with each other, and two cooperators connected by a positive relation both having negative relations with one defector. From a global perspective, the network structure will evolve to become two opposing clusters: cooperators with positive connections and defectors with no edges connected as shown in Fig 5. In Fig 5, although the defectors still want to connect positive edges with cooperators, those cooperators will never give them this chance unless they become cooperators.

### Payoff matrix

Cooperation is modelled in terms of symmetric two-player games. When the model is displayed in a non-signed network, the payoff matrix can be represented as in Table 1. When both players cooperate, they both receive the reward (*R*); when both defect, they both obtain the punishment (*P*); when one cooperates while the other defects, the cooperator suffers the disadvantage of being cheated (*S*), while the defector receives benefit for cheating (*T*) [60]. The PD model assumes *T*>*R*>*P*>*S*, and this model represents the behavior of wanting to connect with cooperators, while keeping a distance from defectors [36–38]. However, this PD model ignores the different relation attributes among players. When we generalize the PD model in signed networks, where signed relations represent cooperative or non-cooperative intentions, the impact of these different signs on individual payoffs should be considered. For example, humans tend to want positive relations with cooperators and negative relations with defectors [1, 43, 46]. The different relation signs have opposite effects on the player’s choice of strategy. To support this rule, negative relations should have opposite effects to positive relations on individual payoffs. Take opportunity cost as an example: if two cooperators are connected by a negative relation, they will lose their chances to get the reward, and thus they will regret being cooperators or connecting a negative line with the other cooperator. The reward can thus be a punishment for their choice. Similarly to other situations in the payoff matrix for negative relations, punishment can also be regarded as a reward for not being punished. As for a negative relation connecting one cooperator and one defector, the defector will lose its chance to receive the benefit of cheating and its opportunity cost is *T*, while the cooperator will get a reward *S* for not being cheated. In this paper, we adopt the payoff matrix for positive relations shown in Table 1 and the payoff matrix for negative relations shown in Table 2.

**Table 1:** Payoff matrix for positive relations.

	C	D
C	*R*	*S*
D	*T*	*P*

**Table 2:** Payoff matrix for negative relations.

	C	D
C	–*R*	–*S*
D	–*T*	–*P*

Santos et al. [36] and Pinheiro et al. [38] proposed a simulation model to analyze the co-evolution of network updating and behavioral updating. However, their model cannot be applied to signed networks. Moreover, the network updating is based on rewiring relations, which may destroy the initial network structure. In reality, it is probably more common for an individual to change a positive (or negative) relation into negative (or positive) (i.e., changing relation signs) than to cut off the relation and establish a new relation with a stranger (i.e., rewiring relations). In this paper, we improve this model as shown in Materials and Methods.

## Materials and methods

Algorithm 1 gives the framework of our computer simulation. Following Pinheiro et al. [38], time proceeds in discrete steps, and at each step every node may be traversed to choose to update its relations or behavioral strategies, and relations update with probability *τ*, while behavioral strategies update with probability (1 − *τ*). Since most of our experimental results become relatively stable (the fraction of cooperators will not increase or decrease more than 10%) within 1,000 iterations, we give the results of 1,000 iterations for every experiment. Moreover, 1,000 iterations is effectively an infinite time, which is much longer than a player’s lifetime. The final results may depend on the simulation rule to some extent, but the framework of the evolutionary game is robust. Here we study the impact of structural balance on the evolutionary dynamics, and we consider three conditions: evolutionary game without structural balance, evolutionary game with structural balance in behavioral updating, and evolution of structural balance where the rules of structural balance are employed in both behavioral and relation updating. Specifically, in pure evolution of structural balance, when updating relations, two players with same the behavioral strategy will set their connection line to be positive, while two players with different behavioral strategies will set their connection line to be negative; when updating behavioral strategies, a player will learn the same strategy from its positive-relation neighbor, but learn the opposite strategy from its negative-relation neighbor.

**Algorithm 1.** The framework of the simulation.

1. Input: The network relation update probability *τ*. The maximum number of iterations *I*_max_. The network size *V*. The intensity of selection *β*.
2. for *g* = 1; *g* ≤ *I*_max_; *g* + +
3.         for *i* = 1; *i* ≤ *V*; *i* + +
4.             if randomly generated *a* < *τ*
5.                 randomly find a neighbor *m* of *i*;
6.                 if the relation sign of *i* and *m* is positive
7.                     if *i* is a cooperator, while *m* is a defector
8.                         *i* has the probability *p* = {1+exp[−*β*(*f*_*i*_−*f*_*m*_)]}^−1^ to change their relation sign into negative;
9.                   elseif *i* and *m* are both defectors
10.                         their relation sign is changed to negative (if in traditional structural balance, their relation sign remains positive);
11.                   end if
12.             else
13.                   if *i* is a defector, while *m* is a cooperator
14.                       *i* has the probability *p* = {1+exp[−*β*(*f*_*i*_−*f*_*m*_)]}^−1^ to change their relation sign into positive (if in traditional structural balance, their relation sign remains negative);
15.         elseif *i* and *m* are both cooperators
16.             their relation sign is changed to positive;
17.         end if
18.       end if
19.     else
20.       randomly find a neighbor *m* of *i*;
21.       if the relation sign of *i* and *m* is positive
22.           *i* has the probability *p* = {1+exp[−*β*(*f*_*m*_−*f*_*i*_)]}^−1^ to learn *m*’s strategy;
23.         else
24.           *i* has the probability *p* = {1+exp[−*β*(*f*_*m*_−*f*_*i*_)]}^−1^ to learn *m*’s strategy (if considering of behavioral structural balance or traditional structural balance, *i* learns *m*’s opposite strategy);
25.           end if
26.         end if
27.     end for
28. end for
29. Output: the updated relations and behavioral strategies.

Relation updating assumes that every individual wants to connect positively with cooperators and negatively with defectors. For a pair of nodes *A* and *B*, if they are both cooperators, their relation tends to be positive; if they are both defectors, their relation tends to be negative; if one of them is a cooperator, while the other is a defector, they have a conflict regarding the relation signs, and the will of *A* prevails with probability *p* = {1+exp[−*β*(*f*_*A*_−*f*_*B*_)]}^−1^, where *f*_*A*_ and *f*_*B*_ are the payoffs accumulated over all interactions with neighbors of *A* and *B*, respectively, and *β* is the intensity of selection [36, 38]. This rule always conforms to some real cases, and if two persons have different choices concerning the same thing, the one who has more capital or more power (e.g., a boss) has more right to make the final decision than the other (e.g., a secretary). However, in the realm of structural balance, the relation between individuals with the same strategies (both cooperators or both defectors) tends to be positive, while that between individuals with different strategies (one is cooperator and the other one is defector) tends to be negative [57].

In updating behavioral strategies, structural balance may conflict with the ideal triangles shown in Fig 2, because it allows the existence of a negative relation connecting one cooperator and one defector, and excludes the negative relation connecting two defectors. We allow two ways to update the behavioral strategy: including structural balance or not. When considering structural balance, if nodes *A* and *B* are connected by a positive link, *A* has probability *p* = {1+exp[−*β*(*f*_*B*_−*f*_*A*_)]}^−1^ to learn *B*’s strategy; while if *A* and *B* are connected by a negative link, *A* has the probability *p* = {1+exp[−*β*(*f*_*B*_−*f*_*A*_)]}^−1^ to learn *B*’s opposite strategy. When not considering structural balance, *A* has the probability *p* = {1+exp[−*β*(*f*_*B*_−*f*_*A*_)]}^−1^ to learn *B*’s strategy, regardless of the sign of their relation.

## Results/Discussion

We use MATLAB to carry out the simulations on a 2.53 GHz CPU and 2.00 GB Memory computer, Windows 7. Consistent with Pinheiro et al. [38], we set the reward for mutual cooperation *R* = 1, the punishment for mutual defection *P* = 0, the cost of being cheated *S* = −*λ*, and the temptation to cheat *T* = 1+*λ*, where 0≤*λ*≤1 represents the dilemma strength in the PD model. By running more simulations, we find that *λ* has little impact on the evolution, and we set *λ* as 0.5 in this paper.

The simulations are carried out on computer generated random networks. Compared with the model in Pinheiro et al. [38], our proposed model requires computing the different situations for different relation signs, and also the level of structural balance, which is much more time costly, so in this paper we choose 100 nodes as the network size for convenience. We have also carried out several simulations on networks with 1,000 nodes and find the conclusions on 100-node networks remain valid for larger network sizes. Although one there could be 150 stable social relationships according to Dunbar's number, just a small fraction of them will participate in a PD game, so we choose Erdős-Rényi random graphs consisting of 100 nodes with average network connectivity 〈*k*〉 = 4 as the experimental networks [61]. The signs “+” or “–” are randomly assigned to these edges; the proportion of “+” edges is *ω* and the proportion of “–” edges is 1 – *ω*. We start with a fraction *γ* of cooperators (1 – *γ* of defectors) randomly distributed in the population.

**Fig 6:**
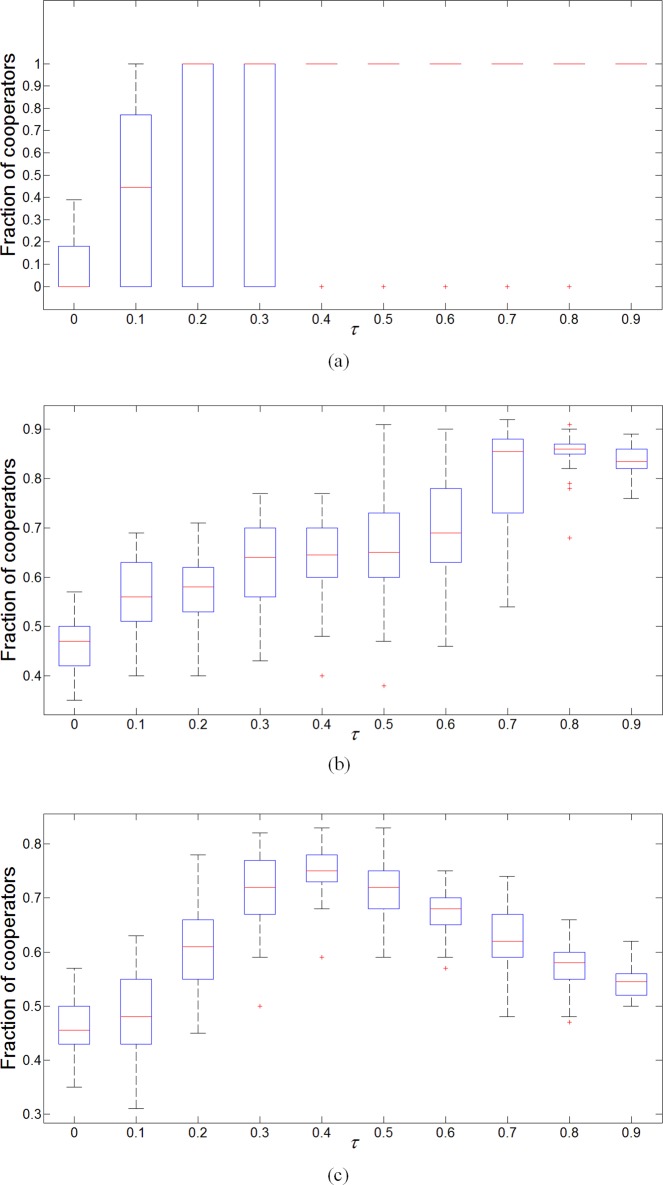
The fraction of cooperators with different *τ* based on different updating methods. The simulation is carried out 10 times on 5 random networks consisting of 100 nodes with average network connectivity 〈*k*〉 = 4. Other parameters are *I*_max_ = 1000, *β* = 10, *ω* = 0.5, *γ* = 0.5 and *λ* = 0.5. The five lines in each box represents the maximum, upper quartile, median, lower quartile and minimum number from top to bottom. The star represents an abnormal value. When the game is played without considering structural balance, the fraction of cooperation will evolve to 0 or 1, and it will have higher probability to evolve to 1 as *τ* increases. When the PD game is played with behavioral updating that considers the structural balance, the fraction of cooperators will also increases as *τ* increases, but will not evolve to 0 or 1. In the pure evolution of structural balance in which both behavioral and edge updating methods consider structural balance, we assign the node attribute ‘+’ as cooperator and set ‘–‘ as defector, and the fraction of cooperators forms an inverted U-type curve as *τ* increases.

Fig 6 shows the fraction of cooperators produced for different values of *τ* based on different updating methods. For *τ* = 0 only behavioral updates are employed in the evolution, while a bigger *τ* represents a faster network adaptation. Among these three methods, the evolutionary game model without structural balance employs both traditional relation and strategy updates, the evolutionary game with structural balance adds the mechanism of structural balance to the strategy updates, while pure evolution of structural balance includes rules of structural balance in both relation and strategy updates. Compared with the pure evolution of structural balance, the fraction of cooperators in evolved social dilemmas increases as *τ* increases, as shown in Fig 6(A) and Fig 6(B). This shows that cooperation prevails when network adaptation is faster in signed networks regardless of the structural balance (i.e., the conclusion proposed by Santos et al. [36] and Pinheiro et al. [38] can be appropriate for signed networks). While in the pure evolution of structural balance, the fraction of cooperators undergoes an inverted U-type change as *τ* increases, as shown in Fig 6(C). On the other hand, it is common for a network to evolve to a structure consisting of all cooperators or all defectors when structural balance is not required, while the fraction of cooperators cannot reach 100% or 0 under the impact of structural balance. In addition, the pure structural balance cannot make players all become cooperators or all become defectors.

**Fig 7:**
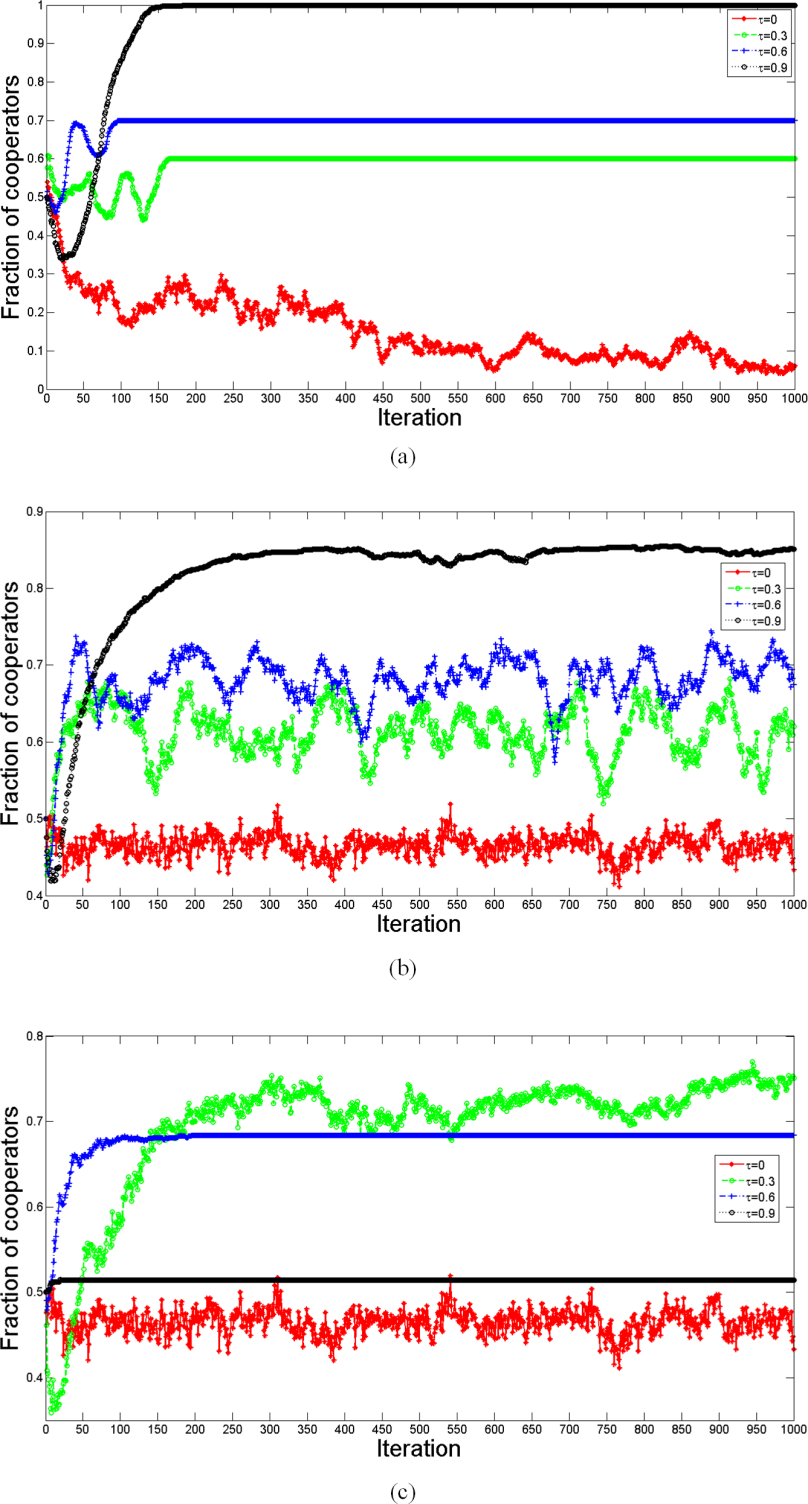
Comparison of the fraction of cooperators for different behavioral updating. The simulation is carried out 10 times on a random network consisting of 100 nodes with average network connectivity 〈*k*〉 = 4. Other parameters are *I*_max_ = 1000, *β* = 10, *ω* = 0.5, *γ* = 0.5 and *λ* = 0.5. Since the curves give average values, most of simulations may evolve to 0 or 1 when not considering of structural balance, and a higher *τ* may guarantee a higher probability for the cooperators evolve to 100%. When considering structural balance in behavioral updating, the curves keep fluctuating, but a higher *τ* generates a higher fraction of cooperators. In pure structural balance evolution, the curves may converge more quickly when *τ* gets bigger, and the fraction of cooperators will increase at first, and then decrease as *τ* increases.

Specifically, we observe the evolution for each iteration of different updating methods. The fraction of cooperators within 1,000 iterations is shown in Fig 7. When not requiring structural balance, the average fraction of cooperation will be higher for larger *τ*. There are some fluctuations for each curve, and the wave length for *τ* = 0.3 and *τ* = 0.6 is greater than that for *τ* = 0.9, which means a smaller *τ* cannot guarantee that the fraction of cooperators increases monotonically. Interestingly, the fraction of cooperation for *τ* = 0.9 undergoes a drop at first and then rebounds to 100% cooperation, which is similar to the results of Pinheiro et al. [38]. Considering structural balance in behavioral updating, Fig 7(B) shows that no curves converge to a fixed value. Since structural balance contradicts the negative relation connecting two defectors, it may force one of the defectors to become a cooperator. However, it also retards achievement of a structure consisting of all cooperators, because it allows a negative relation between one cooperator and one defector, which means it is hard for a defector to become a cooperator when it connects negatively with a cooperator (it first has to transform the negative relation into positive, overcoming the unwillingness of its cooperative neighbor, and then at the next step, it has a chance to become a cooperator). Thus, it is hard for a network to evolve to a structure consisting of all cooperators or all defectors, and it keeps fluctuating around a fixed value. Moreover, we find that a larger *τ* may generate a higher fraction of cooperators and make the evolution fluctuate less. In the pure evolution of structural balance, the curves may converge when *τ* gets larger.

**Fig 8:**
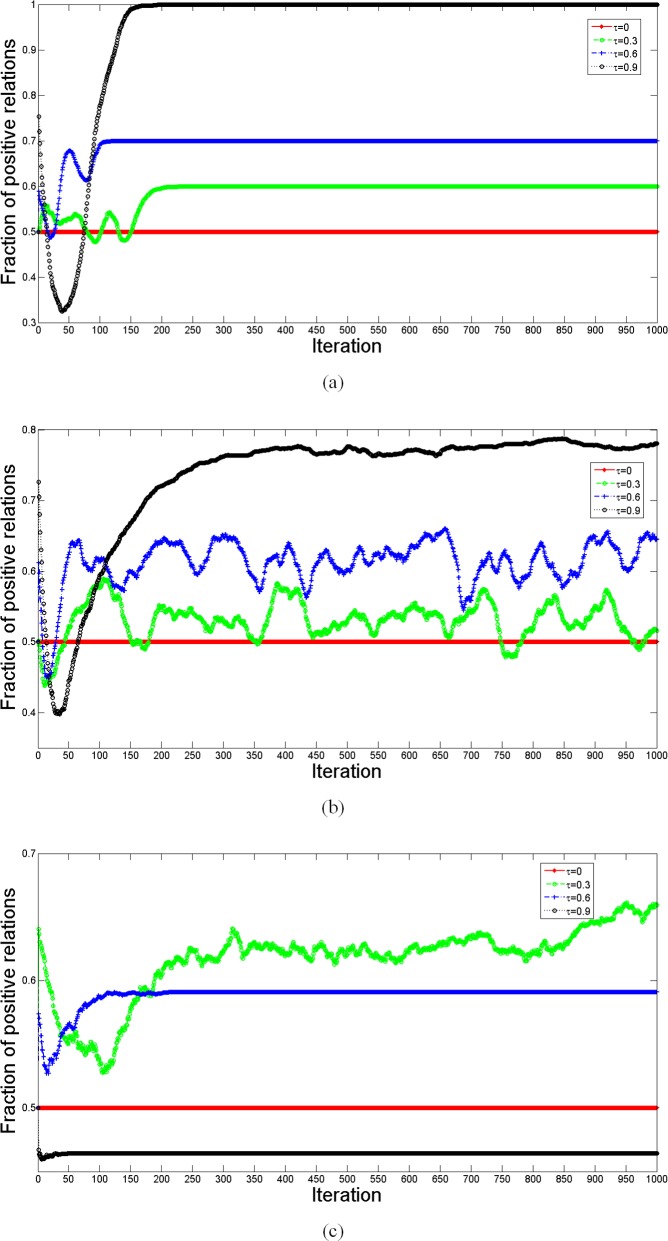
Comparison of the fraction of positive relations for different behavioral updating. The simulation is carried out 10 times on a random network consisting of 100 nodes with average network connectivity 〈*k*〉 = 4. Other parameters are *I*_max_ = 1000, *β* = 10, *ω* = 0.5, *γ* = 0.5 and *λ* = 0.5. Without structural balance, all the network links change to positive links as *τ* = 0.9, but it is hard to transform all the links to positive links when considering structural balance in behavioral updating. Except for the pure evolution of structural balance, positive relations prevail when network adaptation is faster in signed networks. In pure structural balance, positive relations will increase at first, and then decrease as *τ* increases, while the convergence speed will decrease at first, and then increases.

Fig 8 shows the fraction of positive relations corresponding to Fig 7. The fluctuations of the curves in Fig 7 and Fig 8 appear to be synchronous. Therefore, we conclude that positive relations prevail when network adaptation is faster in signed networks, since cooperation supports positive relations.

In this section, we introduce an energy function which is a measure of imbalance in a fully signed network [56, 57, 62]. Eq (1) gives an energy function, where *i*,*j* = 1,2,…,*n* represents two nodes, and *e*_*ij*_∈{1,−1} represents the edge between node *i* and node *j*, *e*_*ij*_ = 1 denotes a positive relation and *e*_*ij*_ = −1 denotes a negative relation; *s*_*i*_∈{1,−1} represents the attribute of node *i*. The value of *H*(*s*) is the level of imbalance, and when *H*(*s*) = 0, the network is balanced.

$$H(s)=\sum_{\left(i,j\right)}\left(1-e_{ij}s_{i}s_{j}\right)/2$$ (1)

**Fig 9:**
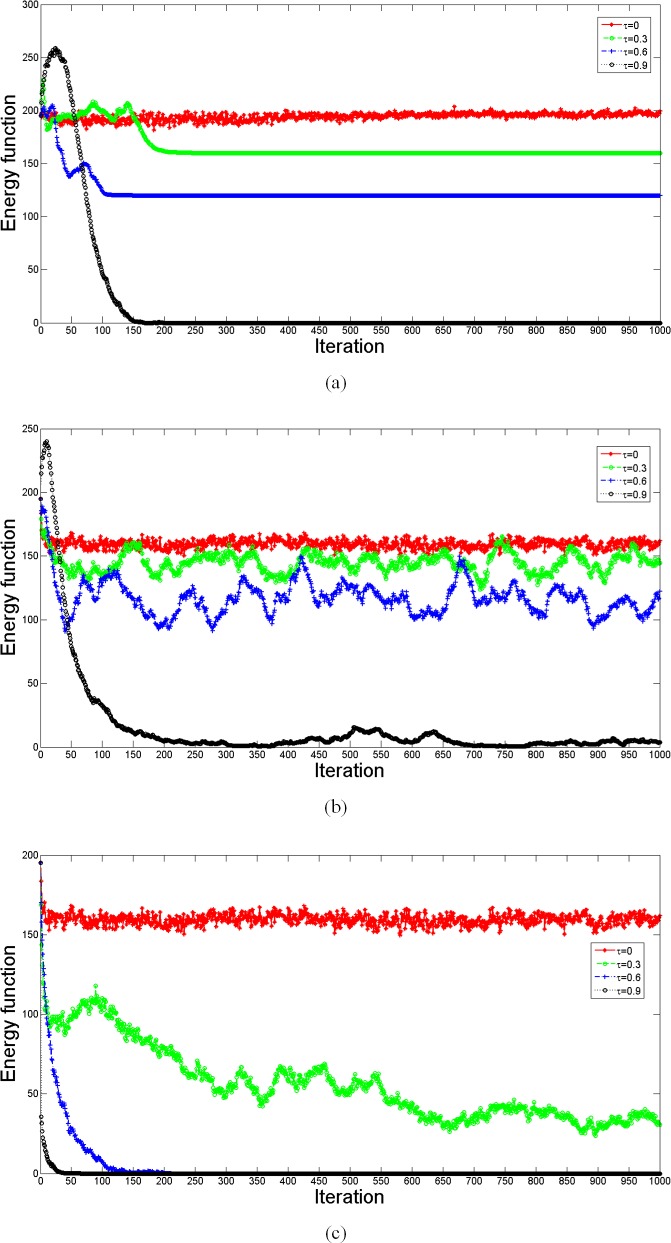
Comparison of the energy function for different behavioral updating. The simulation is carried out 10 times on the random networks consisting of 100 nodes with average network connectivity 〈*k*〉 = 4. Other parameters are *I*_max_ = 1000, *β* = 10, *ω* = 0.5, *γ* = 0.5 and *λ* = 0.5. The energy function represents the imbalanced level of the evolved network. Regardless of the different updating methods, a larger *τ* gives a lower energy function. With behavioral structural balance, the energy function for *τ* = 0.9 declines to 0 at some iteration, and there exist some defectors connected via negative edges to cooperators who are connected by positive edges. In pure structural balance, the curves with *τ* = 0.6 and *τ* = 0.9 evolves to balanced states like the form in Fig 2.

Fig 9 shows the computed energy function corresponding to the simulation in Fig 7. Without structural balance, as shown in Fig 9(A), the curves for *τ* = 0.9 evolve to a balanced state, and all triangles evolve to the form of Fig 3(A), where all players become cooperators connected by positive relations. These calculations confirm our expectation. Considering structural balance in behavioral updating, as shown in Fig 9(B), the curves keep fluctuating, while with *τ* = 0.9, the energy function may decline to 0 after some iterations. It should be noted that the evolved final structure with structural balance is different from that without structural balance, although both evolved structures become balanced with *τ* = 0.9. With behavioral structural balance, there still exist several defectors connected via negative edges to cooperators. In this case, the network evolves to a structure like that in Fig 5, and our discussion is confirmed. In the pure evolution of structural balance, larger *τ* may guarantee a balanced structure for the evolution.

**Fig 10:**
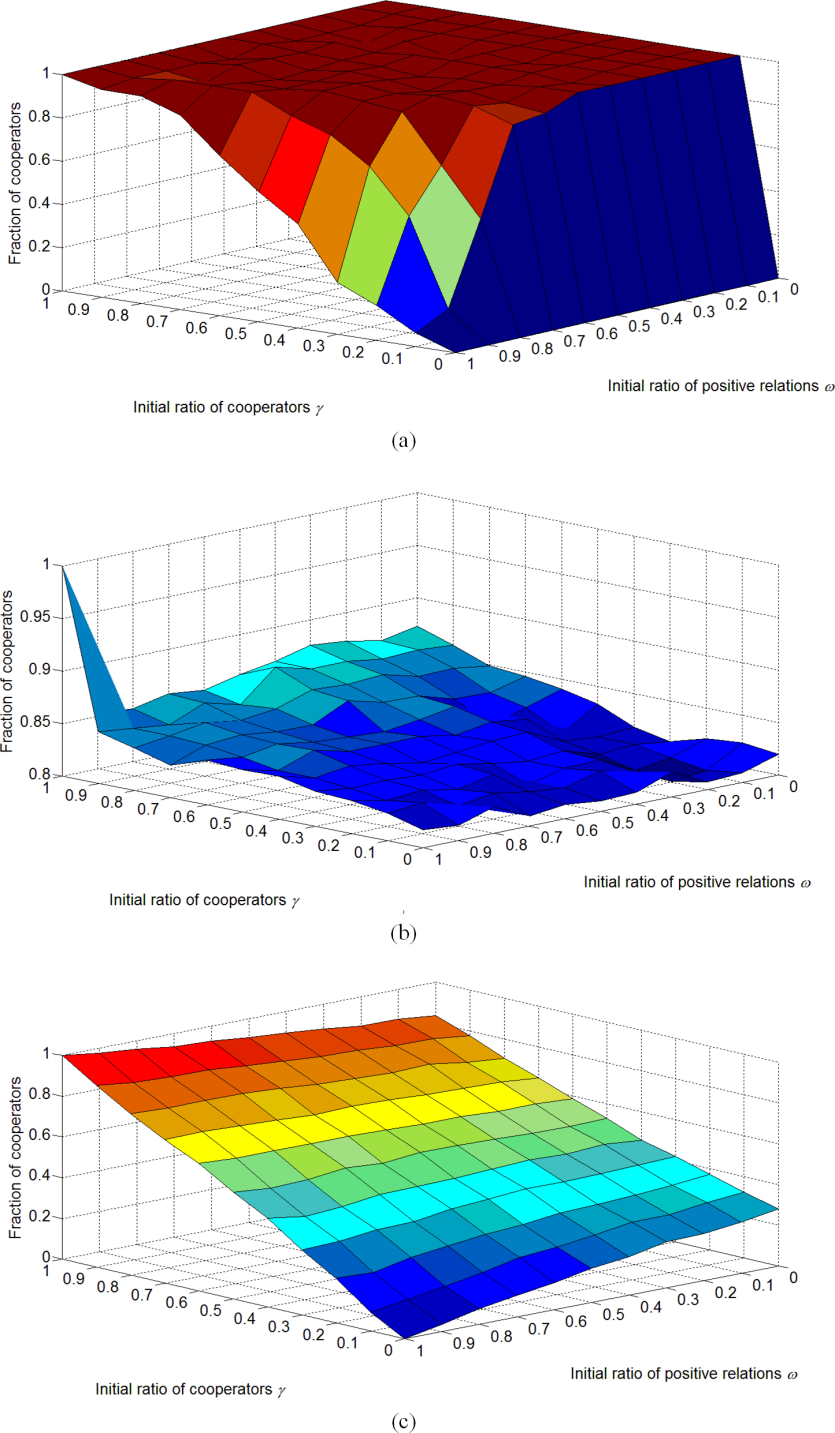
The fraction of cooperators for different *ω* and *γ* based on different behavioral updating. The simulation is carried out 10 times on the random networks consisting of 100 nodes with average network connectivity 〈*k*〉 = 4. Other parameters are *I*_max_ = 1000, *β* = 10 and *λ* = 0.5. Compared with the pure evolution of structural balance, the initial network attributes may have less impact on the evolved cooperation. When not considering structural balance, the fraction of cooperators may decrease as *ω* increases if *ω* ≤ 0.7, while *γ* has a positive impact on the evolution only when *ω* > 0.8. Considering structural balance in the behavioral updating, both *ω* and *γ* have little impact on the evolution. In the pure structural balance, the fraction of cooperators may increase as *γ* increases. It increases as *ω* increases when *γ* > 0.5, but decreases as *ω* increases when *γ* < 0.5.

We also test the effect of some other parameters on the evolution. In order to explore the results at the stable state, we set *τ* = 0.9. Fig 10 shows the simulation results for different *ω* and *γ*; the results with and without structural balance are quite different. Without structural balance, as shown in Fig 10(A), the initial fraction of positive relations, *ω*, has little impact on the evolution when *ω*≤0.7, while the fraction of cooperators may decrease as *ω* increases when *ω*>0.7. This shows that a high fraction of positive relations may retard the increase of cooperators, while negative relations affect cooperation negatively as can be seen from the fraction of cooperators reaching 1 when *ω*≤0.7. On the other hand, the initial fraction of cooperators *γ* has a positive impact on the evolution only when *ω*>0.8. Considering structural balance in the behavioral updating, as shown in Fig 10(B), both *ω* and *γ* have little impact on the evolution. We conclude that the initial attributes of the network have little impact on the evolution of cooperation when considering the effect of structural balance. However, in the pure evolution of structural balance, *ω* and *γ* both have a significant impact on the fraction of cooperators. The fraction of cooperators may increase monotonically as *γ* increases, and it may also increase as *ω* increases when *γ*>0.5, but it may decrease as *ω* increases when *γ*<0.5.

### Conclusion

We have generalized previous models for the dynamic evolution of cooperation in signed networks. We discuss the model of structural balance in networks representing the prisoner’s dilemma (PD), and propose an ideal structure by including both the rules of the PD and structural balance. Further, we propose a new dynamic PD model for signed networks by adjusting both relation signs and behavioral strategies. To analyze the impact of structural balance on the evolutionary dynamics, we study the evolution of the network under three conditions: with or without structural balance in behavioral updating and pure structural balance. Our simulations show that cooperation prevails when individuals have a higher probability of adjusting their relation signs. Structural balance prevents evolution to a structure consisting of 100% cooperators or 100% defectors; instead, it forces the network to evolve to a structure in which cooperators and defectors coexist. Specifically, cooperators connect positively with each other, and negatively to defectors, while no relations exist between defectors. The initial attributes of the network have little impact on the evolution of cooperation without structural balance, but have a strong impact on the pure evolution of structural balance.

Social dilemmas are ubiquitous in human behavior, which makes the PD model applicable in a wide range of natural and social sciences. For example, the evolutionary model has been applied to vaccination, where the dilemma of free riders often occurs [63]. PD models in signed networks provide more ideas to explain such human behaviors. Further, other social mechanisms such as anonymity of opponents [64], adjusted social influence [65] or reciprocity mechanisms [66] can also be included in our proposed model, and we expect that this evolutionary model can facilitate progress for the field of social dilemmas.
