# Correction: The evolution of cooperation in signed networks under the impact of structural balance

**DOI:** 10.1371/journal.pone.0207144

**Published:** 2018-11-02

**Authors:** Xiaochen He, Haifeng Du, Meng Cai, Marcus W. Feldman

There is an error in reference 43. The correct reference is: Righi S, Takács K. Emotional strategies as catalysts for cooperation in signed networks. Advs. Complex Syst. 2014; 17 (02): 1450011–1–1450011–23.

The in-text citation is incorrect in the tenth sentence of the second paragraph in the Introduction section. The sentence should read: Righi and Takács [1, 43, 46] generalized the PD model in signed networks, and first introduced relational signs to summarize affect and emotions between interacting partners.
